# Characterization of Key Metabolic Markers in Hongqujiu Across Different Aging Years Using Metabolomics

**DOI:** 10.3390/jof11050353

**Published:** 2025-05-02

**Authors:** Yiyang Cai, Sunan Yan, Simei Huang, Bin Yang, Wenlan Mo, Lishi Xiao, Xiangyou Li, Zhiwei Huang

**Affiliations:** 1 Engineering Research Centre of Fujian-Taiwan Special Marine Food Processing and Nutrition (Ministry of Education), Fujian Agriculture and Forestry University, Fuzhou 350002, China; cai10404@126.com (Y.C.); huangsm0112@163.com (S.H.); yang_bin2024@163.com (B.Y.); mowenlan2024@163.com (W.M.); xiao_lishi5258@163.com (L.X.); 2 Fujian Key Laboratory of Agro-Products Quality and Safety, Fujian Academy of Agricultural Sciences, Fuzhou 350003, China; yansunan1982@163.com; 3 College of Food Science, Fujian Agriculture and Forestry University, Fuzhou 350002, China; 4 Institute of Quality Standards & Testing Technology for Agro-Products, Fujian Academy of Agricultural Sciences, Fuzhou 350003, China; 5 Fujian Pinghuhong Biological Technology Co., Ltd., Ningde 352256, China; lixiangyou8106@163.com

**Keywords:** Hongqujiu, untargeted metabolomics, volatile compounds, non-volatile compounds, aging years

## Abstract

Hongqujiu, one of the three principal varieties of yellow wine, is a traditional fermented beverage originating from China that employs *Hongqu* as the fermentation agent. In this study, an untargeted metabolomics approach based on gas chromatography–mass spectrometry (GC-MS) and liquid chromatography–mass spectrometry (LC-MS) was applied to systematically analyze the volatile compounds (VOCs) and non-volatile compounds (NVCs) in Hongqujiu across different aging years for the first time. The analysis identified a total of 262 VOCs and 2564 NVCs in samples of Hongqujiu aged for six distinct years. Based on metabolic differences, the samples were categorized into two groups: the low-year group (5-year, 6-year) and the high-year group (8-year, 10-year, 15-year, 20-year). Nineteen VOCs (e.g., 4-amino-butyric acid and diethanolamine) and thirty NVCs (e.g., palmitoylethanolamide and erinacine D) were identified as key differential metabolites distinguishing the low-year group from the high-year group. The higher-year group is enriched with a variety of substances with different flavors or biological activities, such as sugar derivatives, amino acids and their complexes, organic acids and their intermediate metabolites, steroids and terpenoid compounds, lipids and their derivatives, nitrogen-containing heterocycles, and aromatic compounds. The accumulation of these substances not only shapes the unique and rich flavor characteristics of aged red rice wine (such as the caramel aroma and umami peptide flavor), but also endows red rice wine with potential health benefits due to the physiological regulatory functions of some active ingredients. This study contributes to a deeper understanding of the composition and dynamic variations in metabolites in Hongqujiu, offering a scientific foundation for identifying aged Hongqujiu and conducting further research to enhance its quality.

## 1. Introduction

Huangjiu (Chinese yellow wine) is a traditional fermented alcoholic beverage brewed from grains (such as rice, wheat, or millet) using *Jiuqu* (koji) as the fermentation starter. With a documented history spanning millennia, it represents one of the world’s three oldest alcoholic beverages alongside beer and wine, maintaining enduring cultural and scientific significance in East Asian fermentation traditions [1]. *Jiuqu*, the traditional Chinese fermentation starter, is produced through the solid-state fermentation of steamed grains via either artificial inoculation or natural microbial enrichment. It harbors diverse microbial consortia and active enzyme systems that play a crucial role in the fermentation process. Based on the type of grain substrate used, traditional *jiuqu* is primarily classified into two major categories: *Maiqu* (wheat koji), which uses wheat as the main ingredient, and *Miqu* (rice koji), which is based on rice [2]. Traditional Huangjiu can be further classified into three major regional styles based on variations in raw materials and types of *jiuqu* used: Shanghai-style Huangjiu, typified by Shaoxing Huangjiu from Zhejiang Province; Northern-style Huangjiu, exemplified by Jimo Laojiu from Shandong Province; and Southern-style Huangjiu, represented by Hongqujiu from Fujian Province.

Hongqujiu represents one of the most ancient varieties of Huangjiu, traditionally produced through the fermentation of glutinous rice using *hongqu* (red yeast rice) as the primary starter culture. This distinctive alcoholic beverage originates predominantly from southeastern China, with Fujian, Zhejiang, and Guangdong provinces serving as its traditional production centers. *Hongqu* represents a specialized variety of *Miqu*, with Wuyi *Hongqu* and Gutian *Hongqu* being the two predominant types employed as fermentation starters in Hongqujiu production [3]. This traditional fermentation starter is produced via the solid-state fermentation of rice substrates mediated by *Monascus* spp., and has been widely utilized in food fermentation, preservation, and as a natural coloring agent. The uniqueness of *Hongqu* lies in its remarkable biosynthetic capacity to produce specialized metabolites, including natural pigments and bioactive compounds (e.g., monacolin K). This metabolic versatility endows *Hongqu* with dual functionality as both a food ingredient and medicinal substance, making it a paradigmatic representative of traditional fermented products in East Asia [4]. The distinctive feature of Hongqujiu compared to other Huangjiu varieties lies in its use of *Hongqu* as the fermentation starter. During fermentation, *Hongqu* generates a unique and diverse array of microorganisms and metabolites [5,6], which collectively impart Hongqujiu with its characteristic rich and mellow aroma, refreshingly sweet taste profile, and distinctive amber-colored appearance [7,8].

It is commonly stated that “more aging, more flavors”. The term “jiuling” (wine age) functions as an important criterion for classifying most types of Huangjiu, and play a crucial role in quality evaluation as well as consumer purchasing behavior. Generally, with prolonged aging, the flavor profile and overall quality of Huangjiu are substantially enhanced. This improvement is primarily attributed to significant variations in the composition and abundance of microorganisms and their secondary metabolites—including both volatile and non-volatile compounds—generated throughout the fermentation and aging processes [9,10]. Previous studies have shown that in Chinese Huangjiu, the content of compounds such as furans, aldehydes, ketones, esters, acids, and aromatics substances tend to increase substantially during aging. High concentrations of esters, alcohols, and aldehydes enhance the sweetness, alcoholic taste, and fruity aroma of Huangjiu, whereas the decrease in certain sulfides also affects its aroma characteristics [11,12,13].

Metabolomics is a powerful tool for comprehensively characterizing the chemical composition of food and assessing food-quality attributes [14]. When integrated with multivariate statistics analyses, metabolomics effectively reveals key metabolic features and differences among food samples [15]. Wang et al. [16] conducted a non-targeted metabolomics study using GC-MS technology, identifying 100 volatile organic compounds (VOCs), including 33 differential metabolites as aging markers, in Daoguang 25 Baijiu (a classic Chinese *Chenxiang-type* baijiu exclusively aged in century-old wooden container). Their findings revealed distinct volatile profiles across vintages and successfully distinguished six different vintages using chemometric analysis. Similarly, Wang et al. [17] utilized widely targeted metabolomics based on UPLC-ESI-Q TRAP-MS/MS to analyze the non-volatile components of Huangjiu from different regions (Zhejiang Shaoxing Huangjiu, Shandong Jimo Huangjiu, Hubei Fangxian Huangjiu), detecting 1146 non-volatile compounds (NVCs). Another study by Wang et al. [12] applied non-targeted metabolomics using GC-MS to characterize age-dependent changes in VOCs in Chinese Huangjiu, distinguishing samples aged 0–3 years by sulfides, phenols, and small esters and acids, and identifying furans, aromatics, aldehydes, ketones, and most esters and acids as markers for Huangjiu aged 5–15 years. However, studies on Hongqujiu have primarily focused on its microbial communities and fermentation characteristics [18,19], with limited research on the metabolic differences across aging years.

During the fermentation process of *Monascus* (used to produce *Hongqu*), natural harmful substances such as citrinin may be generated [20]. Furthermore, contamination by other molds can lead to the production of toxic compounds. For example, in March 2024, health supplements containing *Monascus* ingredients produced by Kobayashi Pharmaceutical Co., Ltd. (Osaka, Japan). in Japan were contaminated with *Penicillium* during production. This resulted in the formation of compounds such as puberulic acid, posing serious health risks, including kidney disease and even death, to consumers [21]. Therefore, in addition to adhering to strict food-safety standards during the production of fermented foods, rigorous monitoring of safety indicators in end products is essential.

This study focuses on semi-dry Hongqujiu produced by Fujian Pinghu Hong Biotechnology Co., Ltd. (Ningde, China). The metabolic products of Hongqujiu from six different aging periods, including VOCs and NVCs, were analyzed using untargeted metabolomics approaches based on gas chromatography–mass spectrometry (GC-MS) and liquid chromatography–mass spectrometry (LC-MS). This comprehensive comparative analysis of metabolic differences among different aging years of Hongqujiu aims to provide valuable insights into enhancing the quality and safety of Hongqujiu, thereby safeguarding consumer health and well-being.

## 2. Materials and Methods

### 2.1. Samples for Testing

Semi-dry Hongqujiu (Bense-Hongqujiu, BSHQJ) samples with six different aging years (5-year, 6-year, 8-year, 10-year, 15-year, and 20-year) were provided by Fujian Pinghu Hong Biotechnology Co., Ltd. (Ningde, China). The samples were labeled as BS05, BS06, BS08, BS10, BS15, and BS20, respectively, with three replicates for each aging years, resulting in a total of 18 samples.

### 2.2. GC-MS Analysis of Volatile Organic Compounds (VOCs) in Hongqujiu

For VOC extraction, 100 µL of BSHQJ sample was mixed with 300 µL of extraction solution (methanol–acetonitrile = 2:1, containing 5% internal standard *L*-2-chlorophenylalanine). The mixture was vortexed for 30 s, followed by ultrasonic extraction in an ice-water bath for 30 min. The sample was then incubated at −20 °C for 30 min and centrifuged at 13,000× *g* for 15 min at 4 °C. The supernatant was transferred to a glass derivatization vial, dried under nitrogen, and resuspended in 80 µL of methoxamine hydrochloride in pyridine solution (15 mg/mL). After vortexing for 2 min, the mixture was incubated at 37 °C for 90 min to complete the oxime formation reaction. Subsequently, 80 µL of BSTFA (containing 1% TMCS) was added, vortexed for 2 min, and incubated at 70 °C for 60 min. After derivatization, the samples were allowed to equilibrate at room temperature for 30 min before GC-MS analysis.

VOC analysis was conducted using an Agilent 8890B-5977B GC-MS system (Agilent Technologies, Santa Clara, CA, USA) equipped with a DB-5MS capillary column (40 m × 0.25 mm × 0.25 µm, Agilent 122-5532G). One microliter of each sample was injected in split mode (split ratio 10:1) with the inlet temperature set to 300 °C. High-purity helium was used as the carrier gas at a flow rate of 1 mL/min. The oven temperature program started at 60 °C (held for 30 s), increased to 310 °C at a rate of 8 °C/min, and was held at 310 °C for 6 min. Electron impact (EI) ionization was used with the following parameters: transfer-line temperature, 310 °C; ion source temperature, 280 °C; quadrupole temperature, 150 °C; and electron energy, 70 eV. The mass spectrometer was operated in full-scan mode (*m*/*z* 50–500) with a scan frequency of 3.2 scans/s.

### 2.3. LC-MS Analysis of Non-Volatile Compounds (NVCs) in Hongqujiu

For NVC extraction, 200 µL of BSHQJ was mixed with 800 µL of extraction solution (methanol–acetonitrile = 1:1, *v*/*v*) containing four internal standards (e.g., L-2-chlorophenylalanine, 0.02 mg/mL) in a 1.5 mL centrifuge tube. The mixture was vortexed for 30 s and subjected to low-temperature ultrasonic extraction (5 °C, 40 kHz) for 30 min. The samples were then incubated at −20 °C for 30 min and centrifuged at 13,000× *g* for 15 min at 4 °C. The supernatant was transferred to a glass vial, dried under nitrogen, and resuspended in 120 µL of reconstitution solution (acetonitrile–water = 1:1). After vortexing for 30 s, the mixture underwent low-temperature ultrasonic extraction for 5 min (5 °C, 40 kHz), followed by centrifugation at 13,000× *g* for 10 min at 4 °C. The supernatant was transferred into an autosampler vial with an insert for analysis. A pooled quality control (QC) sample was prepared by mixing 20 µL of each sample’s supernatant.

NVC analysis was performed using a UPLC-Triple TOF system (AB SCIEX, Framingham, MA, USA) equipped with an ACQUITY UPLC HSS T3 column (100 mm × 2.1 mm i.d., 1.8 µm; Waters, Milford, CT, USA). The mobile phases consisted of (A) 95% water + 5% acetonitrile (0.1% formic acid) and (B) 47.5% acetonitrile + 47.5% isopropanol + 5% water (0.1% formic acid). The flow rate was set at 0.4 mL/min, with an injection volume of 10 µL, and the column temperature was maintained at 45 °C. Mass spectrometric signals were acquired in both positive and negative ionization modes over a mass range of *m*/*z* 50–1200. The ion spray voltage was set to 5500 V (positive mode) and −4500 V (negative mode). Other parameters included a declustering potential of 80 V, nebulizer and heater gas-flow rates of 50 psi, a curtain gas-flow rate of 35 psi, an ion source temperature of 500 °C, and a collision energy of 40 ± 20 eV.

### 2.4. Data Preprocessing and Analysis

The raw GC-MS data were processed using Mass Hunter Workstation Quantitative Analysis software (v10.0.707.0, Agilent Technologies). Metabolite identification was performed by matching mass spectra against public databases (e.g., NIST, Fiehn, MS-DIAL) and proprietary databases (Majorbio). A three-dimensional matrix in CSV format was generated, containing sample names, metabolite names, and peak areas. Metabolites were identified based on mass spectral matching scores, and differential metabolites were selected using the criteria VIP > 1 and *p* < 0.05.

The raw LC-MS data were processed using Progenesis QI software (v3.0, Waters Corporation) for baseline filtering, peak detection, integration, retention-time correction, and alignment. The resulting data matrix (retention time, peak areas, *m*/*z* ratios, and identification information) was uploaded to the Majorbio Cloud Platform (https://cloud.majorbio.com, accessed on 15 January 2024) for multivariate statistical analysis. Metabolite identification was performed by matching MS and MS/MS spectra against public databases (e.g., HMDB, Metlin) and proprietary databases, with an MS mass error threshold of 10 ppm. Differential metabolites were selected using the same criteria (VIP > 1 and *p* < 0.05).

## 3. Results and Discussion

### 3.1. Results of Volatile Compound Analysis for Six Aging Years of BSHQJ

#### 3.1.1. Detection Results of Volatile Compounds

**Figure 1:**
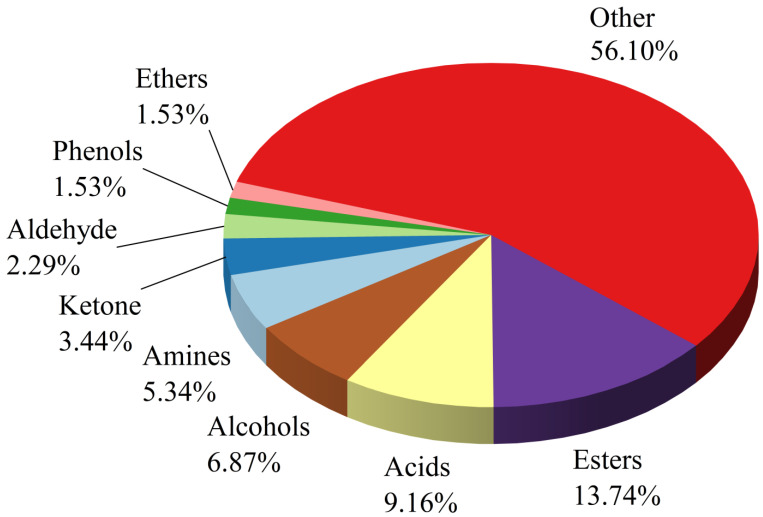
Distribution of volatile compounds in BSHQJ.

To better understand the differences in VOCs between BSHQJ samples of different aging years, this study conducted untargeted metabolomics analysis using GC-MS on six aging years of BSHQJ. A total of 262 VOCs were identified, including 36 esters, 24 acids, 18 alcohols, 9 ketones, 14 amines, 6 aldehydes, 4 phenols, 4 ethers, and 147 other primary and secondary metabolites (Figure 1).

The aging process of BSHQJ involves storing the wine in clay-sealed ceramic jars after undergoing “jianjiu” (high-temperature sterilization treatment). Air slowly permeates through the clay seal, and the microporous structure and large specific surface area of the ceramic jars create a micro-oxygen environment that promotes catalytic oxidation reactions, accelerating the Hongqujiu’s aging process [22].

In this study, in addition to various primary and secondary metabolites, esters, acids, and alcohols were identified as the predominant VOCs in Hongqujiu, consistent with the findings of Hou et al. [23]. *Hongqu*, the exclusive fermentation starter in BSHQJ production, is produced through open fermentation, which gives rise to a complex microbial community that may include filamentous fungi, yeasts, bacteria, and other microorganisms [24]. The unique saccharification and fermentation capabilities of *Hongqu*, combined with synergistic interactions between *Hongqu* and various microorganisms during the brewing process of Hongqujiu, promote the production of enzymes such as saccharase, esterase, and protease. These enzymes contribute to the enrichment of volatile components such as alcohols, esters, aldehydes, and ketones in Huangjiu, thereby enhancing the complexity and depth of its flavor profile [18,25].

Esters are typically derived either from amino acid metabolism or via glycolytic intermediates. *Hongqu* exhibits a significantly higher esterification ability compared to *Yaoqu* and *Xiaomaiqu* [26], which may be a key reason for the high content of esters in BSHQJ. These compounds mainly impart fruity, floral, and sweet flavors to the Huangjiu [27], making it more full-bodied and enhancing its overall flavor. As aging progresses, ester compounds accumulate, adding complexity and richness to the Huangjiu’s aroma [28]. Lactones have been recognized as indicators of aging in wine [29], and this study detected erythronic acid-1,4-lactone across different aging years of BSHQJ. This compound may originate from sugar transformations or interactions between *Monascus* and other microorganisms.

Acid compounds react with ethanol during aging to form aromatic esters, which enhance the richness of Huangjiu, moderate sweetness, and balance the overall flavor by harmonizing with other aroma components [30]. In this study, substances such as pyruvic acid, succinic acid, benzoic acid, and palmitic acid were detected and have been confirmed as the main acid substances in Chinese yellow wine [31]. Additionally, lactic acid, aspartic acid, and l-glutamic acid are the primary amino acids in Huangjiu [32], generally produced during the fermentation and spoilage process of Huangjiu, contributing primarily to its sour and umami flavors [33].

Alcohols are generated through sugar metabolism or amino acid deamination during fermentation and aging. These compounds not only have aromatic properties but also enhance the taste of yellow wine [34]. Key alcohols detected in BSHQJ included phenylethyl alcohol and 2,3-butanediol, the latter being a critical substance for distinguishing Huangjiu of different ages [13]. Phenylethyl alcohol, known for its rose and honey fragrance, is widely used in perfumes and fermented products [35].

Moreover, varieties of aldehydes, phenols, ethers, and other VOCs, along with active ingredients, collectively contribute to the rich flavor profile of BSHQJ. For example, 5-hydroxymethylfurfural, an indicator of non-enzymatic browning, has been reported to possess notable pharmacological properties [36]. 4-ethyl-2-methoxyphenol, which imparts a caramel aroma, and 5-hydroxymethylfurfural have been identified as aging markers in yellow wines [11,37].

To differentiate the six different aging years of BSHQJ samples and reveal their distinct metabolites, multivariate statistical analyses were performed on the 262 VOCs detected by GC-MS.

#### 3.1.2. Cluster Analysis of BSHQJ Samples Based on GC-MS

**Figure 2:**
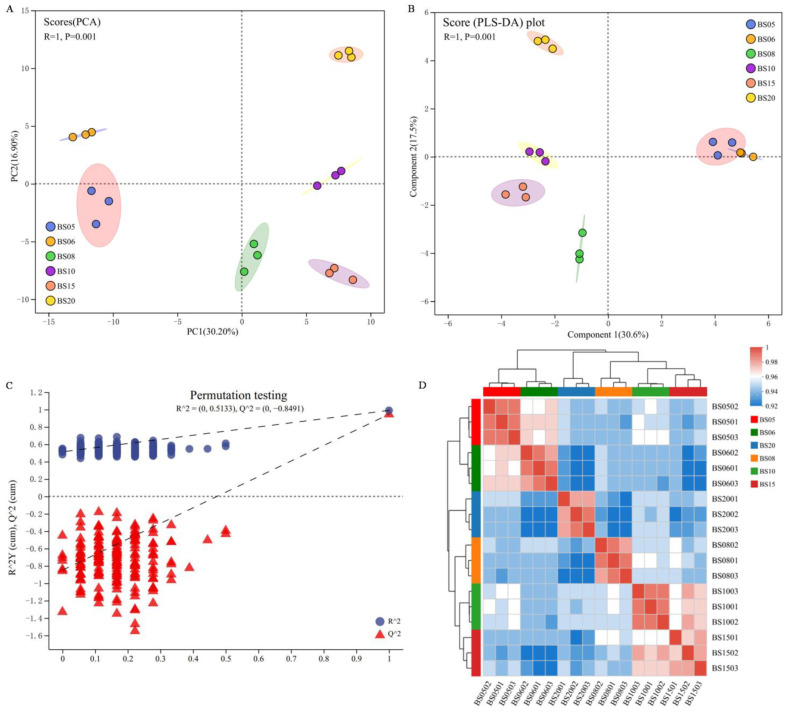
Cluster analysis of BSHQJ samples based on GC-MS. (**A**) PCA plot; (**B**) PLS-DA plot; (**C**) PLS-DA model validation; (**D**) correlation heatmap of BSHQJ samples.

Principal Component Analysis (PCA) and Analysis of Similarities (ANOSIM) were performed to analyze the overall clustering and distribution trends of Hongqujiu samples from different aging years. In the PCA score plot (Figure 2A), the first principal component (PC1) and the second principal component (PC2) accounted for 44.4% of the total variance. The six groups of BSHQJ corresponding to different aging years exhibited distinct separation without overlap, and samples from the same year clustered tightly. Notably, BS05 and BS06 were positioned in close proximity and mainly located in the second quadrant, with their differences from other year samples primarily reflected in PC1. ANOSIM analysis showed that the differences between the different year groups were significantly greater than the differences within the same year group (R = 1, *p* = 0.001).

To further observe differences in characteristic variables between groups, a Partial Least-Squares Discriminant Analysis (PLS-DA) model was constructed. In the PLS-DA score plot (Figure 2B), PC1 and PC2 accounted for 44.7% of the total variance. The clustering effect of PLS-DA outperformed that of PCA, with BS05 and BS06 forming a distinct cluster on the right side along the *Y*-axis. BS08, BS10, and BS15 clustered in the second quadrant, while BS20 was positioned in the third quadrant, showing significant separation from the samples of other years. Model validation using a linear regression test (Figure 2C) confirmed the predictive reliability of the PLS-DA model. When the number of permutations was 200, the model yielded R^2^Y(cum) = 0.5133 and Q^2^(cum) = −0.8491. The positive slopes of the R^2^ and Q^2^ regression lines and a Q^2^Y intercept of less than 0.05 indicated the absence of overfitting.

The above results underscore the pronounced variability in VOC profiles among BSHQJ samples of varying aging years, characterized by distinct clustering patterns and marked aging-related differentiation.

All BSHQJ samples were subjected to Hierarchical Cluster Analysis (HCA). From the correlation heatmap (Figure 2D), it can be observed that wine samples from the same year cluster well and are clearly divided into two groups: the low-year group (BS05, BS06) and the high-year group (BS08, BS10, BS15, BS20). This grouping result aligns with the analyses of PCA and PLS-DA. These findings indicate significant differences in the VOCs among the six aging years of BSHQJ. Specifically, the VOC composition or content of BS05 and BS06 is similar, whereas the VOC composition of BS20 differs substantially from that the wine samples of other years.

#### 3.1.3. Identification and Analysis of Differential VOCs in BSHQJ

##### Comparative Pairwise Analysis of Six Different Aging Years of BSHQJ Based on OPLS-DA

To screen for volatile differential metabolites, pairwise OPLS-DA (Orthogonal PLS-DA) modeling was performed between the six different aging years of BSHQJ (BS06 vs. BS05, BS08 vs. BS05, BS10 vs. BS05, BS15 vs. BS05, BS20 vs. BS05, BS08 vs. BS06, BS10 vs. BS06, BS15 vs. BS06, BS20 vs. BS06, BS10 vs. BS08, BS15 vs. BS08, BS20 vs. BS08, BS15 vs. BS10, BS20 vs. BS10, BS20 vs. BS15). Based on the VIP value (Variable Importance in Projection) of the first principal component in the OPLS-DA model and the T-test *p*-value, differential metabolites were identified. A compound with a VIP > 1 is considered significant for distinguishing the aroma characteristics of different samples. By applying the criteria VIP > 1, *p* < 0.05, and FC > 1, a total of 189 VOCs were identified as differential metabolites between the six different aging years of Hongqujiu.

**Figure 3:**
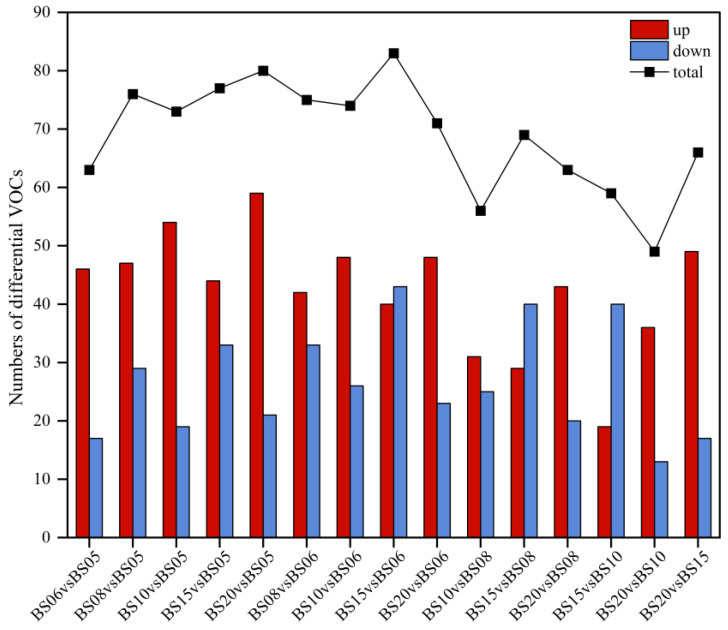
Number of differential VOCs in BSHQJ pairwise-comparison groups for six years.

Based on these 189 VOCs, a comparative analysis of differential VOC counts across aging years was conducted and visualized in a statistical chart (Figure 3). As illustrated by the upper polyline in Figure 3, the number of differential VOCs varies among different comparison groups. A total of 63 VOCs in the low-year group comparisons (BS06 vs. BS05), while the high-year group comparisons (BS10 vs. BS08, BS15 vs. BS08, BS20 vs. BS08, BS15 vs. BS10, BS20 vs. BS10, BS20 vs. BS15) exhibit a range of 56 to 69. However, the comparisons between low-year samples and high-year samples (BS08 vs. BS05, BS10 vs. BS05, BS15 vs. BS05, BS20 vs. BS05, BS08 vs. BS06, BS10 vs. BS06, BS15 vs. BS06, BS20 vs. BS06) show a higher number of differential VOCs, range from 71 to 83. Compared to the differences within the low-year group or high-year group, the number of differential VOCs is significantly higher between the low-year group and high-year group. Additionally, in the majority of pairwise comparisons, the number of upregulated VOCs exceeded that of downregulated VOCs, with the exception of BS15 vs. BS06, BS15 vs. BS08, and BS20 vs. BS15.

These results provide a rationale for classifying BSHQJ into the low-year group (BS05, BS06) and high-year group (BS08, BS10, BS15, BS20) and reveal significant differences in VOCs between these two groups. The differential VOCs may serve as key aroma-related markers distinguishing BSHQJ samples across varying years. Notably, in most pairwise comparisons, higher-year samples exhibited a significantly greater number of VOCs than lower-year samples. This suggests that with increasing aging years, flavor compounds accumulate progressively, contributing to increased flavor complexity. This phenomenon may help explain the traditional notion that “more aging, more flavors.”, as higher-year samples exhibit more complex and diverse VOC profiles, thereby enhancing the complexity and depth of the overall aroma profile.

**Figure 4:**
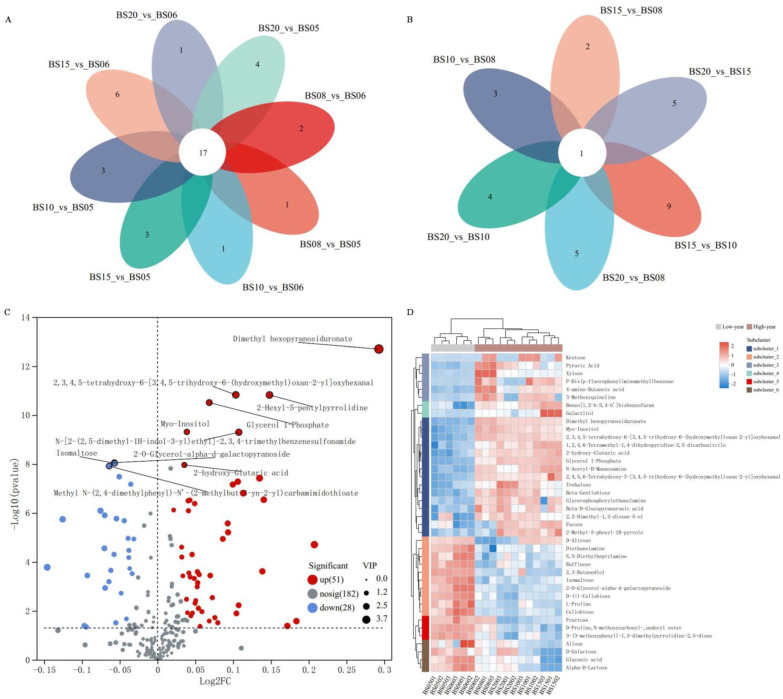
Differential VOC analysis for pairwise comparisons of six aging-year BSHQJ samples. (**A**) Venn diagram of low-year vs. high-year sample comparison groups. (**B**) Venn diagram of comparison groups among high-year samples. (**C**) Volcano plot of differential VOCs between the low-year and high-year groups. (**D**) HCA correlation heatmap of the top 40 differential VOCs in abundance between the low-year and high-year groups.

The Venn diagram analysis (Figure 4A,B) identified 17 common metabolites across pairwise comparisons between low-year and high-year sample groups (Figure 4A), including 2 esters (dimethyl hexopyranosiduronate and alanine, n-methyl-n-butoxycarbonyl-, octyl ester), 2 aldehydes (2,3,4,5-tetrahydroxy-6-[3,4,5-trihydroxy-6-(hydroxymethyl)oxan-2-yl]oxyhexanal and 2,4,5,6-tetrahydroxy-3-[3,4,5-trihydroxy-6-(hydroxymethyl)oxan-2-yl]oxyhexanal), and 1 alcohol (arabitol) which are more abundant in the high-year samples. Additionally, there is only one common metabolite (rhamnose) within the high-year group comparisons (Figure 4B).

##### Comparative Analysis of BSHQJ Between the Low-Year Group and the High-Year Group Based on OPLS-DA

Based on the grouping results in the section of “Comparative Pairwise Analysis of Six Different Aging Years of BSHQJ Based on OPLS-DA”, an OPLS-DA model (high-year vs. low-year) was established between the low-year and high-year groups of BSHQJ for further analysis of the differential VOCs. After 200 permutation tests, the model validation yielded R^2^Y(cum) = 0.996 and Q^2^(cum) = 0.982, with positive slopes for both R^2^ and Q^2^ regression line and the Q^2^Y intercept less than 0.05, indicating excellent predictive ability and no overfitting. Following the criteria VIP > 1, *p* < 0.05, and FC > 1, a total of 79 differential VOCs were identified between the low-year and high-year groups.

Figure 4C shows the volcano plot of differential VOCs between the low-year group and the high-year group, revealing 51 upregulated VOCs and 28 downregulated VOCs. Acid, ester, alcohol, and aldehyde compounds were more abundant in high-year samples (e.g., dimethyl hexopyranosiduronate, myo-inositol, and 2-hydroxy-glutaric acid). In contrast, sugar compounds were more abundant in low-year samples (e.g., isomaltose, cellobiose, d-galactose, and fructose). These results reflect the changes in VOCs during the brewing and aging processes of BSHQJ. In high-year samples, mechanisms such as enzymatic reactions, non-enzymatic reactions, sugar degradation, and amino acid degradation synergistically contribute to the accumulation of esters, alcohols, aldehydes, and other VOCs [30]. The increased presence of acid compounds enhances flavor complexity and imparts distinctive aromatic characteristics. Conversely, low-year samples retain more primary metabolites due to limited aging, resulting in the incomplete transformation of sugar-related compounds. However, these sugars serve as potential precursors for subsequent flavor development during extended aging.

#### 3.1.4. Cluster Analysis of Differential VOCs of BSHQJ in the Low-Year Group and the High-Year Group

To visualize the metabolic trends between the low-year and high-year groups of BSHQJ, a standardized HCA was performed to analyze the relative abundances of the top 40 differential VOCs. The results, shown in Figure 4D, indicate that these 40 differential VOCs are divided into six subclasses. 

**Figure 5:**
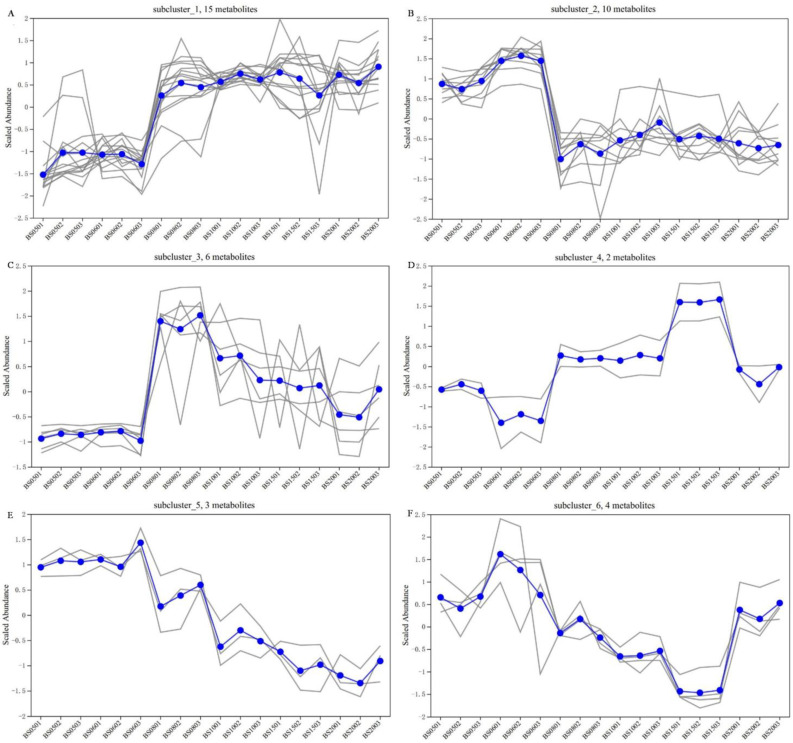
Subclustering trend diagram of six subclusters of VOCs. (**A**) First subcluster; (**B**) second subcluster; (**C**) third subcluster; (**D**) fourth subcluster; (**E**) fifth subcluster; (**F**) sixth subcluster. In each subgraph, each grey line represents an individual metabolite, while the blue line indicates the average expression level across all metabolites in each sample.

Based on the subcluster trend plots of the six subclusters (Figure 5), the analysis shows that in subclusters 1, 3, and 4, most VOCs have lower content in the low-year group compared to the high-year group. Conversely, in subclusters 2, 5, and 6, most VOCs have higher content in the low-year group, with the majority being sugar compounds (such as cellobiose, isomaltose, raffinose, and fructose). The content of 2,3-butanediol in subcluster 2 is highest in the low-year group samples, which is consistent with the findings of Yu et al. [13]. Fucose in subcluster 1, isomaltose, and raffinose in subcluster 2 are positively associated with the years of the high-year group BSHQJ. Fucose has been confirmed to play an important role in the taste characteristics of yellow wine [32]. Other key compounds—such as dimethyl hexopyranosiduronate (subcluster 1), 4-amino-butanoic acid (GABA), and 3-methoxyquinoline (subcluster 3)—were significantly more abundant in the high-year group, with GABA content lowest in the low-year group and peaking in BS08, a pattern consistent with the observations of Zhao et al. [10]. GABA is a bioactive compound with physiological functions such as lowering blood pressure and regulating blood lipids [38]. It is synthesized through the decarboxylation of glutamate catalyzed by glutamate decarboxylase (GAD). Notably, glutamate is one of the primary amino acids produced during Hongqujiu fermentation [39]. As the aging years of BSHQJ increase, notable shifts occur in the composition of its microbial community and enzyme substances [40], which may lead to the accumulation of certain functional substances in the high-year group samples, potentially inhibiting the synthesis of GABA. Additionally, some microbes may further utilize GABA during aging, converting it into secondary metabolic products, resulting in relatively reduced GABA content in the high-year group samples [41].

A further analysis of the relationship between each subcluster VOC and the six aging years indicates that in subcluster 1, three VOCs (2-hydroxy-glutaric acid, glycerol 1-phosphate, and myo-inositol) are positively correlated with the wine samples’ years. In contrast, in subcluster 5, three VOCs (d-proline,n-methoxycarbonyl-,undecyl ester, 3-(3-methoxyphenyl)-1,3-dimethylpyrrolidine-2,5-dione, and fructose) are negatively correlated with the wine samples’ years. N-acetyl-d-mannosamine (subcluster 1), benzo[1,2-b:5,4-b’]bisbenzofuran and galactitol (subcluster 4) were significantly more abundant in BS15 compared to other years, with galactitol exhibiting negligible levels in all samples except BS15. However, in subcluster 6, three VOCs (d-galactose, gluconic acid, and alpha-d-lactose) exhibit significantly lower contents in BS15 compared to other years.

In summary, in subcluster 1, beta-gentiobiose, 2,4,5,6-tetrahydroxy-3-[3,4,5-trihydroxy-6-(hydroxymethyl)oxan-2-yl]oxyhexanal, 2,3,4,5-tetrahydroxy-6-[3,4,5-trihydroxy-6-(hydroxymethyl)oxan-2-yl]oxyhexanal, myo-inositol, n-acetyl-d- mannosamine, dimethyl hexopyranosiduronate, and glycerol 1-phosphate, in subcluster 3, 4-amino-butanoic acid, pyruvic acid, 3-methoxyquinoline, p-bis(p-fluorophenyliminomethyl)benzene, and in subcluster 4, benzo[1,2-b:5,4-b’]bisbenzo-furan, are significantly lower in the low-year group compared to the high-year group. In subcluster 2, isomaltose, cellobiose, d-(+)-Cellobiose, 2-o-glycerol-alpha-d-galactopyranoside, and diethanolamine, and in subcluster 5, 3-(3-methoxyphenyl)-1,3-dimethylpyrrolidine-2,5-dione, d-proline,n-methoxycarbonyl-,undecyl ester, are significantly higher in the low-year group compared to the high-year group. These VOCs can serve as key differential VOCs for distinguishing between the low-year and high-year groups of BSHQJ.

### 3.2. Results of Non-Volatile Compound Analysis for Six Aging Years of BSHQJ

#### 3.2.1. Detection Results of Non-Volatile Compounds

**Figure 6:**
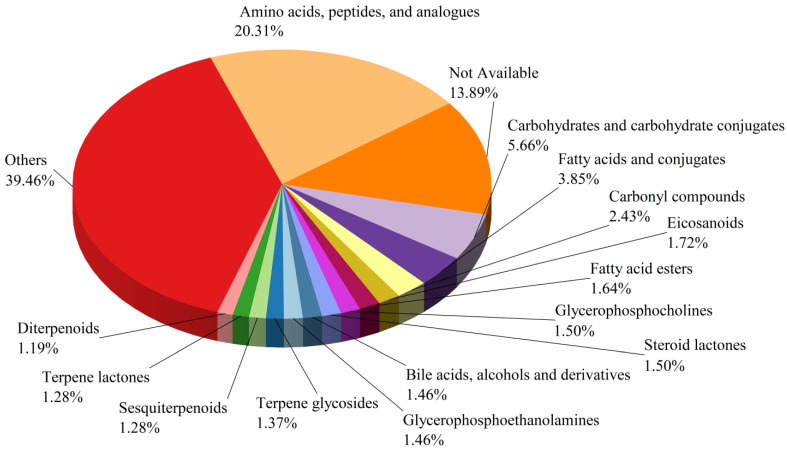
Distribution of non-volatile compounds in BSHQJ.

In the LC-MS analysis, both positive and negative ion modes were used, detecting a total of 2564 NVCs. The metabolites were classified into subclasses in the HMDB database, including 459 amino acids, peptides, and analogs, 314 not available, 128 carbohydrates and carbohydrate conjugates, 87 fatty acids and conjugates, 55 carbonyl compounds, 39 eicosanoids, 37 fatty-acid esters, 34 glycerophosphocholines, 34 steroid lactones, 33 bile acids, alcohols, and derivatives, 33 glycerophos-phoethanolamines, 31 terpene glycosides, 29 sesquiterpenoids, 29 terpene lactones, 27 diterpenoids, and 892 other compounds (Figure 6).

Huangjiu is rich in bioactive components such as amino acids, vitamins, phenolic compounds, oligosaccharides, trace elements, and mineral elements, which provide various health benefits, including cholesterol reduction, antioxidation, antihypertension, and antibacterial properties [42,43]. Studies show that during the brewing process of Hongqujiu, proteases produced by *Hongqu* and various microbes can break down proteins, thereby significantly increasing the content of amino acids and polypeptides in Huangjiu. This not only enhances the flavor of Huangjiu but also improves its nutritional value and functional characteristics [8,44]. This study detected various amino acids and polypeptides from different aged wine samples, including gamma-glutamylthreonine, citrulline, serylarginine, histidylasparagine, asparaginyl-lysine, and tryptophyl-serine. These substances contribute to different flavor characteristics such as bitterness (e.g., valine, phenylalanine), umami (e.g., aspartic acid and glutamic acid), and sweetness (e.g., alanine and proline), giving BSHQJ a rich and mellow unique taste [33,45]. Futhermore, the organic acids in BSHQJ include caprylic acid, valeric acid, propionic acid, lactic acid, and palmitic acid, which not only help enhance the flavor of Hongqujiu but may also play a positive role in alleviating obesity and diabetes-related diseases [17,46,47]. Li et al.’s research indicates that compared to *Baiqu* rice wine’s NVCs, palmitic acid is a unique NVC for Hongqujiu [48].

Polyphenols have strong antioxidant properties. Isoferulic acid, detected in BSHQJ samples, exhibits anti-inflammatory, antiviral, antioxidative, and antidiabetic characteristics [49]. Functional sugars are considered “dietary fiber” due to their low sweetness and low-calorie attributes [50]. Gentiobiose, lentinan, chitobiose, d-arabitol, xylobiose, and other functional sugars in BSHQJ not only possess antioxidant capabilities but also provide various health benefits such as antitumor effects, obesity improvement, and enhancement of intestinal barrier function [51,52,53,54]. Biogenic amines are widely present in fermented foods. While moderate intake benefits human health, excessive intake may pose toxic risks. Biogenic amines detected in BSHQJ include beneficial components such as tyramine and dihydroergotamine, which regulate the nervous system and enhance immunity [55], as well as compounds associated with potential toxic effects, such as histamine and spermidine [56].

To distinguish the six different aging years of BSHQJ and reveal their differential metabolites, multivariate statistical analysis was conducted on the 2564 NVCs detected by LC-MS.

#### 3.2.2. Cluster Analysis of BSHQJ Samples Based on GC-MS

**Figure 7:**
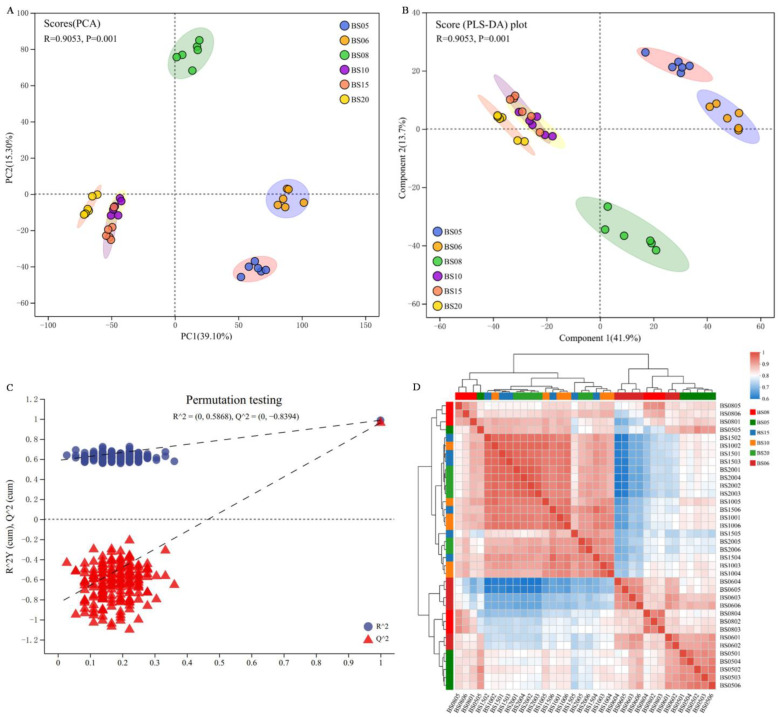
Cluster analysis of BSHQJ samples based on LC-MS. (**A**) PCA plot; (**B**) PLS-DA plot; (**C**) PLS-DA model validation; (**D**) correlation heatmap of BSHQJ samples.

In Figure 7A, the PCA plot shows that PC1 accounts for 37.6%, and PC2 accounts for 14.7%. An overlap between BS10 and BS15 suggests minimal differences in their NVC compositions, indicating compositional similarity between these two aging years. Except for BS08, the samples from other years are mainly distributed below the *Y*-axis, indicating that the NVC composition of BS08 has significant differences from other samples. Meanwhile, there were significant differences among all different-year BSHQJ sample groups (ANOSIM: R = 0.9053, *p* = 0.001). In Figure 7B, the PLS-DA plot shows that PC1 accounts for 40.2%, and PC2 accounts for 13.5%. The separation trend of BSHQJ from different aging years in the PLS-DA plot mirrors the distribution pattern observed in the PCA plot. The model validation results (Figure 7C) indicate that the model has effective predictive ability and does not exhibit overfitting.

The HCA results (Figure 7D) show that, compared to the VOCs analysis results in Section 3.1.2, the clustering of NVCs for BS10, BS15, and BS20 is more significant. This observation aligns with previous PCA and PLS-DA analysis results. In an integrated analysis of PCA, PLS-DA, and HCA analyses, the NVC composition of BS08 has certain differences from the high-year group, so it can be classified into the low-year group. Accordingly, based on the analysis results of NVC differences, BS05, BS06, and BS08 are classified into the low-year group, while the high-year group includes BS10, BS15, and BS20.

#### 3.2.3. Identification and Analysis of Differential NVCs in BSHQJ

##### Comparative Pairwise Analysis of Six Different Aging Years of BSHQJ Based on OPLS-DA

**Figure 8:**
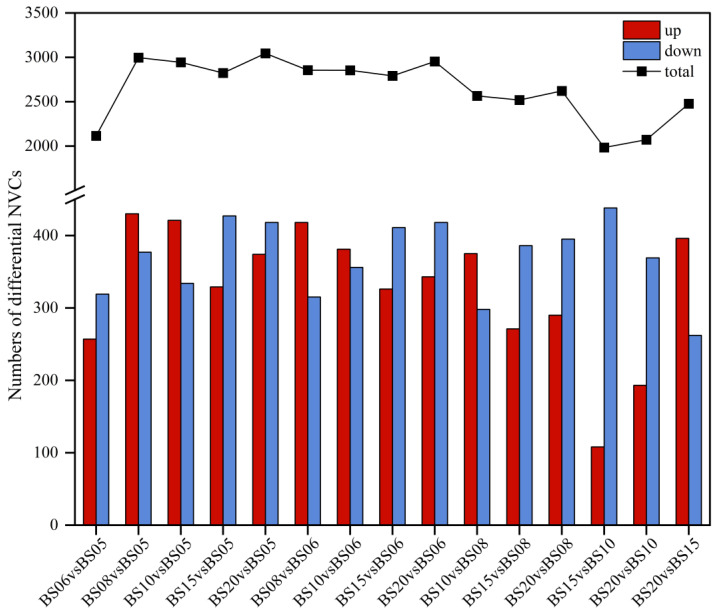
Number of differential NVCs in BSHQJ pairwise-comparison groups for six years.

To better screen differential NVCs, paired OPLS-DA models were established between the six different aging years of BSHQJ (BS06 vs. BS05, BS08 vs. BS05, BS10 vs. BS05, BS15 vs. BS05, BS20 vs. BS05, BS08 vs. BS06, BS10 vs. BS06, BS15 vs. BS06, BS20 vs. BS06, BS10 vs. BS08, BS15 vs. BS08, BS20 vs. BS08, BS15 vs. BS10, BS20 vs. BS10, BS20 vs. BS15). Based on VIP > 1, *p* < 0.05, and FC > 1 criteria, a total of 1894 differential NVCs were identified between the paired comparing groups of different aging years. As indicated by the upper polyline in the statistical chart of differential NVCs among different vintage samples (Figure 8), the number of differential NVCs between the low-year group comparison (BS06 vs. BS05) and some high-year group comparisons (BS15 vs. BS10, BS20 vs. BS10, BS20 vs. BS15) is slightly lower than that of other comparison groups. This indicates that the composition or content of NVCs in BS05 and BS06 are similar, and the metabolic composition among BS10, BS15, and BS20 is also relatively similar. This result is consistent with the analysis results of PCA and PLS-DA. Compared with BS06 vs. BS05, BS08 vs. BS05, BS15 vs. BS08, and BS20 vs. BS08, the number of differential NVCs in BS08 vs. BS05 and BS08 vs. BS06 is significantly higher. This indicates that there are significant differences in NVCs between BS08 and BS05 and BS06. Therefore, BS08 should be reclassified into the high-year group. Such a grouping scheme (the low-year group includes BS05, BS06; the high-year group includes BS08, BS10, BS15, BS20) is more reasonable and can better reflect the significant differences between the two groups.

**Figure 9:**
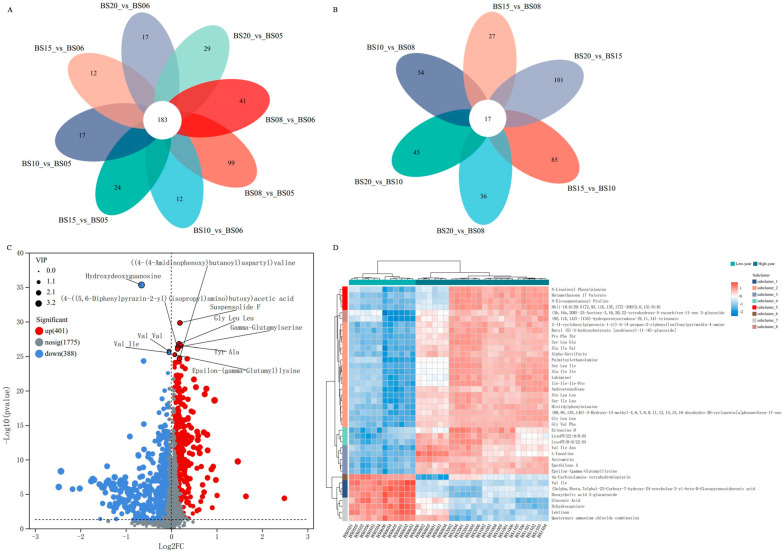
Differential NVC analysis for pairwise comparisons of six aging-year BSHQJ samples. (**A**) Venn diagram of low-year vs. high-year sample comparison groups. (**B**) Venn diagram of comparison groups among high-year samples. (**C**) Volcano plot of differential NVCs between the low-year and high-year groups. (**D**) HCA correlation heatmap of the top 40 differential NVCs in abundance between the low-year and high-year groups.

Venn diagram analysis revealed 183 common NVCs between the low-year samples and high-year samples paired when comparing groups (Figure 9A), while only 17 common NVCs within the high-year group (Figure 9B).

##### Comparative Analysis of BSHQJ Between the Low-Year Group and the High-Year Group Based on OPLS-DA

Based on the grouping results of 3.2.3.1, an OPLS-DA model (High-year vs. Low-year) was established between the low-year group and high-year group of BSHQJ to further analyze the differential NVCs between the two groups. The model yielded R^2^Y(cum) = 0.99 and Q^2^(cum) = 0.985 following 200 permutation tests. The slopes of the R^2^ and Q^2^ regression lines are both positive, and the Q^2^Y intercept is less than 0.05, indicating that the model has good predictive ability and no overfitting occurs. Based on VIP > 1, *p* < 0.05, and FC > 1 criteria, a total of 789 differential NVCs were identified between the low-year group and high-year group.

Figure 9C is a volcano plot of differential NVCs between the low-year group and the high-year group, with a total of 401 upregulated NVCs and 388 downregulated NVCs. Among them, amino acids, peptides, and their analogs exhibit higher abundance in both groups, with even higher content in the high-year group. The number of upregulated differential NVCs is slightly greater than that of the downregulated ones, indicating that the high-year-group wine samples have a more abundant profile of NVCs. Additionally, most of the top ten differential NVCs ranked by *p* value belong to upregulated metabolites. This suggests that BSHQJ is rich in components such as amino acids and their derivatives, which tend to increase with prolonged aging.

#### 3.2.4. Cluster Analysis of Differential NVCs of BSHQJ in the Low-Year Group and the High-Year Group

Using standardized HCA, the relative contents of the top 40 differential NVCs were analyzed, with the results shown in Figure 9D. These 40 differential NVCs were classified into eight subclasses.

**Figure 10:**
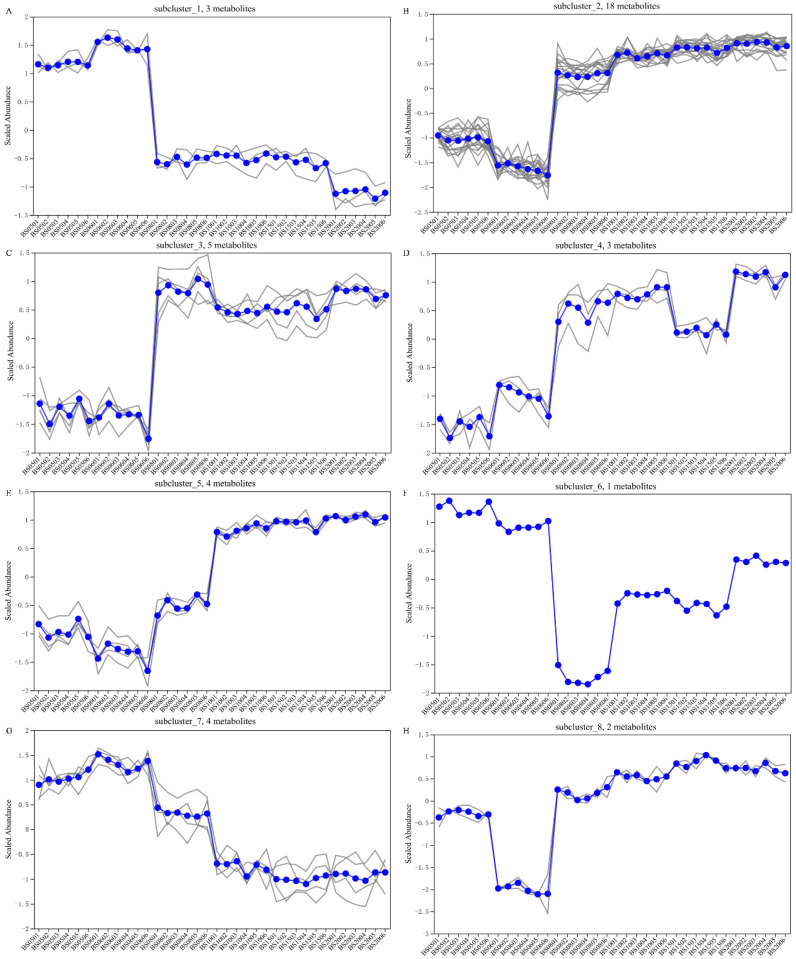
Subclustering trend diagram of six subclusters of NVCs. (**A**) First subcluster; (**B**) second subcluster; (**C**) third subcluster; (**D**) fourth subcluster; (**E**) fifth subcluster; (**F**) sixth subcluster; (**G**) seventh subcluster; (**H**) eighth subcluster. In each subgraph, each grey line represents an individual metabolite, while the blue line indicates the average expression level across all metabolites in each sample.

Based on the subcluster trend plots of the 8 subclusters (Figure 10), the analysis indicates that in subclusters 1, 6, and 7, most NVCs exhibited significantly higher levels in the low-year group than in the high-year group. Conversely, in subclusters 2, 3, 4, and 5, most NVCs have significantly lower contents in the low-year group than in the high-year group. Among them, subclusters 2 and 3 include 13 amino acid peptides (Ala Leu Leu, Ser Leu Ile, Gly Leu Leu, histidylphenylalanine, Ser Ile Leu, Ser Ile Leu, Gly Val Phe, Ala Ile Val, Pro Phe Thr, Ser Leu Glu, Ala Ile Ile, Ile-Ile-Ile-Pro, Val Ile Asn, epsilon-(gamma-glutamyl)lysine, l-canaline). These substances exhibit certain antioxidant activities and contribute to flavors such as umami, bitterness, and sweetness [45]. This may be the reason why the high-year group wines have higher nutritional value and a richer taste profile.

A further analysis of the trends of NVCs in each subcluster within the low-year and high-year groups revealed that most NVCs in subcluster 7 are negatively correlated with the years in the high-year group, while most NVCs in subclusters 2, 5, 6, and 8 are positively correlated with the years in the high-year group. In subcluster 2, the metabolite palmitoylethanolamide (PEA), derived from palmitoylethanolamine, is a lipid-derived signaling compound used to treat chronic pain, inflammation, and certain brain diseases [57]. Additionally, in subcluster 6, only one NVC (4a-carbinolamine tetrahydrobiopterin) has significantly higher content in the low-year group than in the high-year group, with the lowest content observed in BS08. Subcluster 8 includes two NVCs ((3b,16a,20R)-25-acetoxy-3,16,20,22-tetrahydroxy-5-cucurbiten-11-one 3-glucoside, (9Z,11E,14Z)-(13S)-hydroperoxyoctadeca-(9,11,14)-trienoate), belonging to triterpenoid compounds and fatty-acid derivatives, respectively. Both exhibit the lowest content in BS06, with no significant difference in content across the high-year group. In subcluster 4, NVCs show an upward trend in the low-year group but have the lowest content in BS15 within the high-year group. Among them, the diterpene compound erinacine D exhibits activity in stimulating nerve growth factor [58]. Subcluster 5 NVCs have the highest content in the higher-year wines of the high-year group (BS10, BS15, BS20), with no significant differences among these three years.

In summary, in subcluster 1, Val Ile, (3alpha,5beta,7alpha)-23-carboxy-7-hydroxy-24-norcholan-3-yl-beta-d-glucopyranosiduronic acid, and deoxycholic acid 3-glucuronide; in subcluster 7, quaternary ammonium chloride combinations have significantly higher contents in the low-year group than in the high-year group. In subcluster 2, (8R,9S,13S,14S)-3-hydroxy-13-methyl-4,6,7,8,9,11,12,14,15,16-decahydro-3H-cyclopenta[a]phenanthren-17-one, lubiminol, androstenedione, palmitoylethanolamine, Ala Leu Leu, Ser Leu Ile, Gly Leu Leu, histidylphenylalanine, Ser Ile Leu, Gly Val Phe, Ala Ile Val, butyl (s)-3-hydroxybutyrate [arabinosyl-(1->6)-glucoside], Pro Phe Thr, 2-(4-cyclohexylpiperazin-1-yl)-5-(4-propan-2-ylphenyl)sulfonylpyrimidin-4-amine, Ser Leu Glu, alpha-guttiferin, Ala Ile Ile, Ile-Ile-Ile-Pro; in subcluster 3, astromicin, epothilone A, Val Ile Asn, epsilon-(gamma-glutamyl)lysine, l-canaline; in subcluster 4, lysoPE(22:0/0:0), lysoPE(0:0/22:0), and erinacine D all have significantly lower contents in the low-year group compared to the high-year group. These NVCs can serve as key differential NVCs for distinguishing between the low-year and high-year groups of BSHQJ.

## 4. Conclusions

Using untargeted metabolomics techniques based on GC-MS and LC-MS, this study systematically analyzed both the volatile and non-volatile components of BSHQJ for the first time. Through GC-MS detection, a total of 262 VOCs were identified, including 36 esters, 24 acids, and 18 alcohols, among others. A total of 189 differential VOCs between six different aging years of BSHQJ were screened. Multivariate statistical analysis divided the six different aging years of BSHQJ into the low-year group (BS05, BS06) and high-year group (BS08, BS10, BS15, BS20). Further screening identified 79 differential VOCs between the two groups, among which 19 metabolites could serve as key differential VOCs to distinguish between the low-year and high-year groups of BSHQJ: beta-gentiobiose, 2,4,5,6-tetrahydroxy-3-[3,4,5-trihydroxy-6-(hydroxymethyl)oxan-2-yl]oxyhexanal, 2,3,4,5-tetrahydroxy-6-[3,4,5-trihydroxy-6-(hydroxymethyl)oxan-2-yl]oxyhexanal, myo-inositol, n-acetyl-d-mannosamine, dimethyl hexopyranosiduronate, glycerol 1-phosphate, 4-amino- butanoic acid, pyruvic acid, 3-methoxyquinoline, p-bis(p-fluorophenyliminomethyl)benzene, benzo[1,2-b:5,4-b’]bisbenzofuran, isomaltose, cellobiose, d-(+)-cellobiose, 2-o-glycerol-alpha-d-galactopyranoside, diethanolamine, 3-(3-methoxyphenyl)-1,3-dimethylpyrrolidine-2,5-dione and d-proline,n-methoxycarbonyl-,undecyl ester.

A total of 2564 NVCs were detected through LC-MS, and 1894 differential NVCs were screened by pairwise-comparison analysis of six different aging years of BSHQJ. Multivariate statistical analysis divided the six different aging years of BSHQJ into the low-year group (BS05, BS06) and high-year group (BS08, BS10, BS15, BS20). Further screening identified 789 differential NVCs between the two groups, among which 30 metabolites could serve as key differential NVCs to distinguish between the low-year and high-year groups of BSHQJ: Val Ile, (3alpha,5beta,7alpha)-23-carboxy-7-hydroxy-24-norcholan-3-yl-beta-d-glucopyranosiduronic acid, deoxycholic acid 3-glucuronide, quaternary ammonium chloride combination, (8R,9S,13S,14S)-3-hydroxy-13-methyl-4,6,7,8,9,11,12,14,15,16- decahydro-3h-cyclopenta[a]phenanthren-17-one, lubiminol, androstenedione, palmitoylethanolamine, Ala Leu Leu, Ser Leu Ile, Gly Leu Leu, histidylphenylalanine, Ser Ile Leu, Gly Val Phe, Ala Ile Val, Butyl (s)-3-hydroxybutyrate [arabinosyl-(1->6)-glucoside], Pro Phe Thr, 2-(4-cyclohexylpiperazin-1-yl)-5-(4-propan-2-ylphenyl)sulfonylpyrimidin-4-amine, Ser Leu Glu, alpha-guttiferin, Ala Ile Ile, Ile-Ile-Ile-Pro, astromicin, epothilone A, Val Ile Asn, epsilon-(gamma-glutamyl)lysine, l-canaline, lysoPE(22:0/0:0), lysoPE(0:0/22:0), and erinacine D.

The results of this study indicate that the aging duration has a significant influence on the compositional diversity of Hongqujiu. Compared with samples with shorter aging periods, Hongqujiu with extended aging undergoes more comprehensive and synergistic biochemical transformations during maturation. These include enzymatic reactions (such as glycosidic bond hydrolysis and esterification), non-enzymatic browning processes (such as Maillard reactions and caramelization), and macromolecular degradation (such as polysaccharide cleavage and amino acid decarboxylation/deamination). Collectively, these processes contribute to the accumulation of a broader spectrum of potentially beneficial metabolites in long-aged Hongqujiu. These metabolites comprise a broad spectrum of structurally diverse compounds exhibiting distinct flavor characteristics and/or biological activities, including sugar derivatives (e.g., beta-gentiobiose, 2,4,5,6-tetrahydroxy-3-[3,4,5-trihydroxy-6-(hydroxymethyl)oxan-2-yl]oxyhexanal, 2,3,4,5-tetrahydroxy-6-[3,4,5-trihydroxy-6-(hydroxymethyl)oxan-2-yl]oxyhexanal, myo-inositol, dimethyl hexopyranosiduronate), amino acids and their complexes (e.g., l-canaline, Ala Leu Leu, Ser Leu Ile, Gly Leu Leu, Histidylphenylalanine, Ser Ile Leu, Gly Val Phe, Ala Ile Val, Pro Phe Thr, Ser Leu Glu, Ala Ile Ile, Ile-Ile-Ile-Pro, Val Ile Asn, epsilon-(gamma-glutamyl)lysine), organic acids and their metabolic intermediates (e.g., glycerol 1-phosphate, 4-amino-butanoic acid, pyruvic acid), steroids and terpenoids (e.g., (8R,9S,13S,14S)-3-hydroxy-13-methyl-4,6,7,8,9,11,12,14,15,16-decahydro-3H-cyclopenta[a]phenanthren-17-one, lubiminol, androstenedione, alpha-guttiferin, erinacine D), lipids and their derivatives (e.g., palmitoylethanolamine, lysoPE(22:0/0:0), lysoPE(0:0/22:0)), as well as nitrogen-containing heterocycles and aromatic compounds (e.g., 3-methoxyquinoline, p-bis(p-fluorophenyliminomethyl)benzene, benzo[1,2-b:5,4-b’]bisbenzofuran). The accumulation of these substances not only shapes the unique and rich flavor characteristics of aged red yeast wine (such as caramel aroma and umami peptide flavor), but also gives it potential healthcare value due to the physiological regulation function of some active ingredients. For instance, GABA exerts antihypertensive effects and inhibits lipid absorption through neuromodulation; myo-inositol regulates glucose and lipid metabolism via insulin signaling pathways, thereby contributing to metabolic homeostasis, while steroidal compounds may mediate metabolic regulation through estrogen receptors. Consequently, the synergistic accumulation of these bioactive metabolites not only enhances the organoleptic quality of Hongqujiu, but also provides theoretical support for its potential health-promoting functions, particularly in terms of antioxidant activity and metabolic regulation.

In summary, this study utilized untargeted metabolomics techniques to systematically analyze the composition characteristics of VOCs and NVCs in Hongqujiu from different aging years, revealing their variation patterns with aging years and identifying key differential metabolites that can distinguish between low-year and high-year Hongqujiu. The results not only provide a scientific basis for the aging grouping of Hongqujiu but also lay a significant theoretical foundation for further exploration of its aging mechanism and the accumulation pattern of flavor substances. In future studies, it is essential to investigate the specific functional mechanisms of these key differential metabolites during the brewing and aging processes of Hongqujiu, which will offer theoretical support for optimizing the brewing process and enhancing the quality of Hongqujiu.

## Data Availability

All data generated or analyzed during this study are included in this article.

## Abbreviations

BSHQJ, Bense-Hongqujiu; GC-MS, gas chromatography–mass spectrometry; LC-MS, liquid chromatography–mass spectrometry; VOC, volatile compound; NVC, non-volatile compound; PCA, Principal Component Analysis; PLS-DA, Partial Least-Squares Discriminant Analysis; OPLS-DA, orthogonal PLS-DA; HCA, Hierarchical Cluster Analysis.
